# Cytomegalovirus Infection in Ireland: Seroprevalence, HLA Class I Alleles, and Implications

**DOI:** 10.1097/MD.0000000000002735

**Published:** 2016-02-12

**Authors:** Jaythoon Hassan, Derek O’Neill, Bahman Honari, Cillian De Gascun, Jeff Connell, Mary Keogan, David Hickey

**Affiliations:** From the National Virus Reference Laboratory, University College Dublin, Belfield, Dublin (JH, CDG, JC); National Histocompatibility and Immunogenetics Service for Solid Organ Transplantation, Beaumont Hospital, Dublin (DON, MK); Centre for Support and Training in Analysis and Research, Department of Public Health, Physiotherapy and Population Science, University College Dublin, Belfield, Dublin (BH); and Transplantation Unit, Beaumont Hospital, Dublin, Ireland (DH).

## Abstract

Cytomegalovirus (CMV) infections occur worldwide and primary infection usually occurs in early childhood and is often asymptomatic whereas primary infection in adults may result in symptomatic illness. CMV establishes a chronic latent infection with intermittent periods of reactivation. Primary infection or reactivation associate with increased mortality and morbidity in those who are immunocompromised. Transplacental transmission may result in significant birth defects or long-term sensorineural hearing loss.

We performed a study to determine the CMV seroprevalence and the association between HLA Class I alleles and frequency of CMV infection in Ireland. The presence of CMV IgG, a marker of previous CMV infection, was determined for a cohort of 1849 HLA typed solid organ transplant donors between 1990 and 2013. The presence of CMV IgG was correlated with HLA type.

The CMV seroprevalence in solid organ transplant donors was 33.4% (range 22–48% per annum) over the time period 1990 to 2013. Multivariate logistic regression analysis showed that both age and HLA alleles were associated with CMV seropositivity. A significant and positive relationship between age and CMV seropositivity was observed (OR = 1.013, *P* < 0.001, CI [1.007, 1.019]). Chi-square analysis revealed that the female gender was independently associated with CMV seropositivity (*P* < 0.01). Seroprevalence in women of reproductive age (20–39 years) was significantly higher than men of the same age (37% vs 26%, *P* < 0.01). The frequencies of HLA-A1, HLA-A2, and HLA-A3 in our cohort were 40.8%, 48.8%, and 25.9%, respectively. Logistic regression analysis showed that the presence of HLA-A1 but not HLA-A2 or HLA-A3 was independently associated with CMV seronegativity (*P* < 0.01). Interestingly, individuals who co-expressed HLA-A2 and HLA-A3 alleles were significantly more likely to be CMV seropositive (*P* < 0.02). The frequencies of HLA-B5, HLA-B7, and HLA-B8 in our cohort were 6.1%, 31.2%, and 30.8%, respectively. The presence of the most common inherited haplotype in the Irish population, HLA-A1, B8 was significantly associated with CMV seronegativity (OR = 1.278, *P* < 0.001, CI [1.049, 1.556]).

CMV seroprevalence is lower in Ireland compared with other countries. The high frequency of HLA-A1 in the Irish population may, in part, have a role in the reduced susceptibility to CMV infection.

## INTRODUCTION

Cytomegalovirus (CMV) is a member of the herpes virus family. It is a 235-kb double-stranded linear DNA virus and is transmitted via saliva, urine, and most other bodily fluids of infected individuals. In addition, CMV can be transmitted through infected organs and blood products. This ease of transmission results in particularly high seroprevalence in certain parts of the world. However, seroprevalence differs greatly from country to country ranging from 50% up to 90% depending on many factors including ethnicity and socio-economic status.^1^ Primary CMV infection gives rise to lifelong latency and reactivation can occur.

Congenital cytomegalovirus infection, which has an occurrence of between 0.2% and 2.5% worldwide, may result from primary infection, reactivation of maternal CMV infection, or reinfection with another CMV strain. Primary infection is associated with increased morbidity and mortality for the fetus.

Publications describing CMV seroprevalence have generally restricted the population cohorts studied to pregnant women. A meta-analysis among women of reproductive age showed that globally CMV seroprevalences ranged from 40% to 100%. ^1^ The highest seroprevalence rates were observed in Africa, Asia, and South America and the lowest rates were observed in Western Europe and North America.^1^ A study examining pregnant women in Ireland in 2002 found that CMV seroprevalence in Irish women at 30.4% was significantly lower than that in non-Irish pregnant women (89.7%) who were mostly from Sub-Saharan Africa, Eastern Europe, and Asia.^2^

CMV infection in immunocompetent individuals is usually asymptomatic, although symptomatic infection is more common as age at primary infection increases. CMV IgG seropositivity is considered the best laboratory measure of past CMV infection. However, cell-mediated immunity is more important than humoral immunity in controlling CMV infection.^3^ Cellular immune responses measured as the in vitro production of IFN-γ by CD8+ T lymphocytes in response to CMV peptides has recently been defined as a biomarker associated with the risk of CMV infection and disease post-transplant.^4–7^ As these cellular immune responses are restricted by HLA Class I alleles for peptide recognition, in addition to determining the CMV seroprevalence rate in Ireland, the association of CMV seropositivity with HLA Class I alleles was also examined.

## MATERIALS AND METHODS

### Study Population

This study was performed on a cohort of 1849 solid organ transplant donors in Ireland from 1990 to 2013. Data was collated by the National Histocompatibility and Immunogenetics Service for Solid Organ Transplantation, at Beaumont Hospital, Dublin. Ethical approval for the study was obtained from the Human Research Ethics Committee, University College Dublin.

### Determination of Anti-CMV IgG Antibodies

Pretransplant serological testing for CMV IgG was performed. During the early years, the Biotest anti-HCMV recombinant IgG test (Biotest AG, Dreieich, Germany), a solid phase enzyme-linked assay (EIA), was utilized in accordance with the manufacturer's recommendations. Subsequently, the Abbott Architect CMV IgG assay (Abbott Diagnostics), a chemiluminescent microparticle immunoassay (CMIA), for the detection of anti-HCMV IgG was performed.

### Determination of HLA Class I Alleles

DNA typing of HLA Class I alleles was performed using the LABType^®^ SSO Typing method (one Lambda Inc, USA). Briefly, target DNA was amplified using biotinylated group-specific primers; this allowed it to be detected using R-Phycoerythrin-conjugated Streptavidin. The amplicon was denatured and hybridized to complementary DNA probes that were conjugated to fluorescently coded microspheres. A flow analyzer was used to identify the PE intensity on each microsphere and assignment of HLA type was based on the reaction patterns associated with published HLA gene sequences.

### Statistical Analysis

Logistic regression was performed to determine significance of association between various HLA-A and HLA-B Class I alleles to CMV seronegativity.

Descriptive statistical analysis for CMV seropositivity was performed. Chi-square analysis was performed to test the association between gender and CMV seropositivity. Hardy–Weinberg equilibrium was used to assess the frequency of the HLA alleles by the chi-square test. All statistical tests were performed with SPSS 22 at a significance value of 5%.

## RESULTS

**FIGURE 1:**
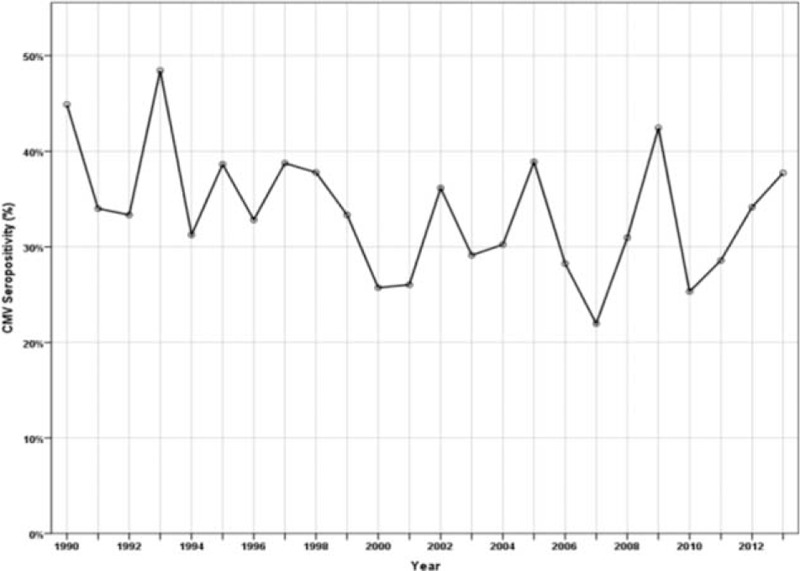
CMV seroprevalence in solid organ transplant donors, 1990 to 2013.

**FIGURE 2:**
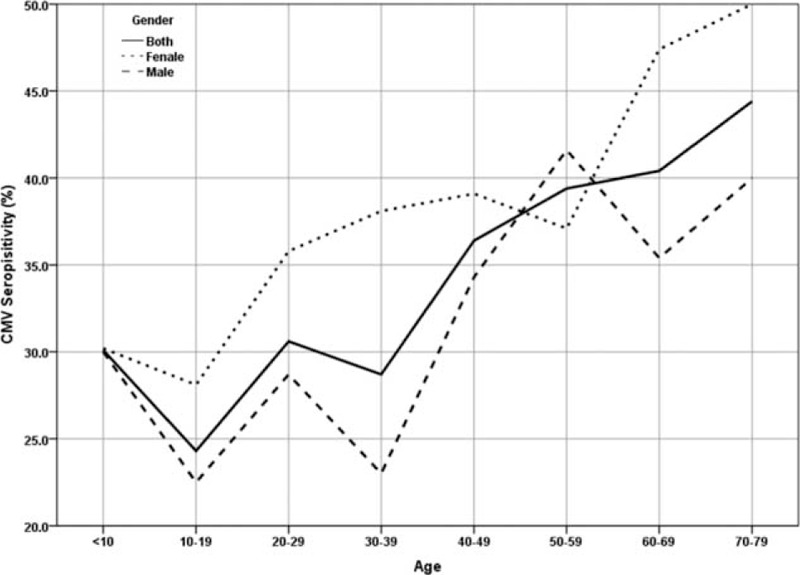
Association of CMV seropositivity with age and gender.

The CMV seroprevalence in solid organ transplant donors ranged from 22% to 48% over the time period 1990 to 2013 with a mean seroprevalence of 33.4% (Figure 1). In the 1990s, the mean seroprevalence was 37.3% with a peak in 1993 at 48.4%. The period from 2000 to 2010 showed a lower mean seroprevalence at 30.5% with a peak of 42.5% in 2009. The lowest CMV seroprevalence was observed in 2007 at 22%. CMV seroprevalence in the solid organ transplant donors increased steadily with age (Figure 2). In the 10 to 19 years age group, CMV seroprevalence was 24.3%. However, only when individuals were aged >60 years a seroprevalence of >40% observed.

The frequencies of HLA-A1, HLA-A2, and HLA-A3 in our cohort were 40.8%, 48.8%, and 25.9%, respectively. For 5 HLA alleles, HLA A1, A3, B5, B7, and B8, the fit to Hardy–Weinberg equilibrium (all *P* values < 0.05) and for HLA A2 (*P* values >0.2) was observed. Logistic regression analysis showed that the presence of HLA-A1 but not HLA-A2 or HLA-A3 was independently associated with CMV seronegativity (*P* < 0.01) (Table 1). Interestingly, individuals who co-expressed HLA-A2 and HLA-A3 alleles were significantly more likely to be CMV seropositive (*P* < 0.02). The frequencies of HLA-B5, HLA-B7, and HLA-B8 in our cohort were 6.1%, 31.2%, and 30.8% respectively. The presence of HLA-B5 was associated with increased prevalence of seropositivity (OR = 1.79, *P* = 0.003). The presence of the most common inherited haplotype in the Irish population, HLA-A1, B8 was significantly associated with CMV seronegativity (OR = 0.783, *P* = 0.015) (Table 1).

**TABLE 1:**
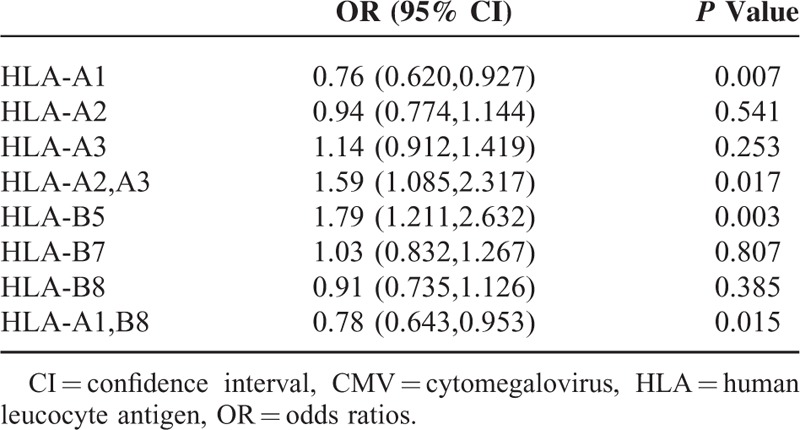
Association of HLA-A and HLA-B Class I Alleles With CMV Seronegativity

**FIGURE 3:**
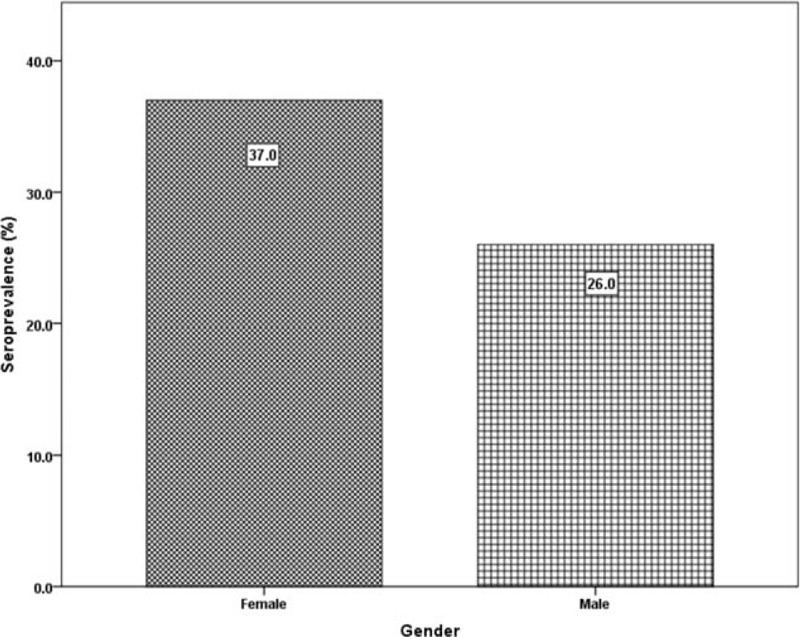
CMV seropositivity is significantly higher in women of reproductive age.

The results of logistic regression showed a significant and positive relationship between age and CMV seropositivity (OR = 1.013, *P* < .001, CI[1.007, 1.019]). Chi-square analysis revealed that female gender was independently associated with CMV seropositivity (*P* < 0.01). Overall women showed a higher seroprevalence compared to men in all age cohorts studied except 50 to 59 years. Seroprevalence in women of reproductive age (20–29 and 30–39 years) was significantly higher than men of the same age (35.8% vs 28.7% and 38.1% vs 23%, *P* < 0.01) (Figure 3).

## DISCUSSION

The present study provides an analysis of the association of age, gender, and HLA alleles with CMV seropositivity. Although many studies have reported on age-specific CMV seroprevalence, information on the HLA allele association is lacking. In 1849 solid organ transplant donors, we observed that CMV seroprevalence is significantly lower in Ireland (33.4%) compared to other countries such as USA (50.4%),^8^ Australia (57%),^9^ and Portugal (77%).^10^ Irish pregnant women were reported to have a seroprevalence rate of 30.4% which was lower than the 56.3% previously reported in pregnant women from other Western European nations.^2^

In agreement with other studies, we found that the CMV seroprevalence rate increased with age. The seropositivity level in donors < 10 years of age was 30.1% and this increased to 44.4% in donors of 70 to 79 years of age. When data was analyzed according to gender, both men and women of <10 years of age had a similar seroprevalence of 30.0% and 30.2%, respectively. However, in the 70 to 79 years of age, seroprevalence levels in women were higher at 50% compared to men at 40.0%. Several studies have confirmed the higher CMV seropositivity rates in women compared to men^1^ and this is especially important for women of reproductive age and probably reflects the increased exposure of women to CMV secreted in the urine of young children during child care. A limitation of the present study is that confounding factors including race/ethnicity, socioeconomic status, interaction with young children were not recorded and therefore could not be examined.

As development of adaptive immunity to CMV by CD8+ T cells is HLA Class I restricted, we investigated the association of CMV seronegativity, and therefore inherent protection against CMV, with HLA Class I alleles. The most common inherited haplotype present in the Irish population is HLA-A1, B8, DR3 which occurs at a frequency of 16% to 20%.^11^ A previous study of 10,000 bone marrow donors showed that 43% expressed the HLA-A1 allele^12,13^ and this is in agreement with our finding of 40.8% in solid organ transplant donors. In the present study, frequencies of HLA-A2 and A3 were 48.8% and 25.9% respectively in our cohort. Of note, logistic regression analysis revealed that the presence of HLA-A1 but not HLA-A2 or A3 was significantly independently associated with CMV seronegativity. This finding in the Irish population may, in part, have a role in the reduced susceptibility to CMV infection. Further evidence to support our findings comes from a recent study which calculated an overall incidence of 0.23% for congenital CMV in Ireland.^14^ This is lower than the prevalence estimate of 0.7% based on 15 studies with a total of 117,986 infants screened.^15^ Dollard and colleagues ^15^ found that the overall prevalence of congenital CMV infection in industrialized countries is likely to be 0.6% to 0.7% which the authors state is more precise than the range of 0.2% to 2.5% often cited in the literature. Another study in agreement with our findings reported that the incidence of CMV infection and CMV disease were low in a cohort of Irish liver transplant recipients ^16^ compared with the published literature.^17,18^ However, future studies in other countries are required to confirm this association and to address the strong selection pressure which HLA genes are under due to mechanisms such as selection, mutation, genetic drift but in particular migration.
