# Cytomegalovirus Infection in Ireland: Seroprevalence, HLA Class I Alleles, and Implications: Erratum

**DOI:** 10.1097/01.md.0000484983.28067.b9

**Published:** 2016-06-24

In the article “Cytomegalovirus Infection in Ireland: Seroprevalence, HLA Class I Alleles, and Implications”,^1^ which appeared in Volume 95, Issue 6 of *Medicine*, Dr. Honari's degree was incorrectly listed as MSc. Dr. Honari has a PhD. The article has since been corrected online.
